# Seismic ground vibrations give advanced early-warning of subglacial floods

**DOI:** 10.1038/s41467-020-15744-5

**Published:** 2020-05-19

**Authors:** Eva P. S. Eibl, Christopher J. Bean, Bergur Einarsson, Finnur Pàlsson, Kristin S. Vogfjörd

**Affiliations:** 1 Institute of Geosciences, University of Potsdam, Potsdam, Germany; 2 School of Earth Sciences, University College Dublin, Belfield, Dublin Ireland; 3 Geophysics Section, School of Cosmic Physics, Dublin Institute for Advanced Studies, Merrion Square, Dublin Ireland; 4 Icelandic Meteorological Office, Bústaðavegi 7–9, 108 Reykjavík Iceland; 5 Institute of Earth Sciences, University of Iceland, Askja, Building of Natural Sciences, Sturlugata 7, 101 Reykjavík Iceland

**Keywords:** Natural hazards, Geophysics, Seismology

## Abstract

Glacier runoff and melt from volcanic and geothermal activity accumulates in glacier dammed lakes in glaciated areas around the world. These lakes eventually drain, creating hazardous subglacial floods that are usually only confirmed after they exit the glacier and reach local river systems, which can be many tens of kilometres from the flood source. Once in the river systems, they travel rapidly to populated areas. Such delayed detection represents a potentially lethal shortcoming in early-warning. Here we demonstrate how to advance early-warning potential through the analysis of four such floods in a glaciated region of Iceland. By comparing exceptional multidisciplinary hydrological, GPS and seismic ground vibration (tremor) data, we show that array analysis of seismic tremor can be used for early location and tracking of the subglacial flood front. Furthermore the timing and size of the impending flood can be estimated, prior to it entering the river system. Advanced warnings of between 20 to 34 hours are achieved for large (peak discharge of more than 3000 m^3^/s, accumulation time of ~ 5.25 years) to small floods (peak discharges from 210 to 380 m^3^/s, accumulation times of ~ 1.3 years) respectively.

## Abstract (web summary)

Subglacial lakes and jökulhlaups (glacier outburst floods) are common in volcanic and glaciated environments, and can pose potential threats to communities living downstream. Here, the authors find that seismic tremor signals during subglacial floods can be used to locate and track the speed and size of the flood before it arrives at the river system, and improves previous methods of early glacial flood warning by a factor of 5.

## Introduction

Glaciers worldwide can host subglacial or dam marginal lakes. These lakes are fed by rivers or accumulate water from ice or snow melt, precipitation or geothermal melting^1–4^. Ultimately the accumulated water drains as a flood, which can be hazardous to people, property and infrastructure. For example, a flood in 1981 in Nepal with an estimated peak discharge exceeding 2000 m^3^/s led to extensive loss of human life, infrastructure and arable land^5^. The volcanically triggered flood in 1996 in Iceland reached a discharge of 50,000 m^3^/s causing severe damage to infrastructure^6^. As subglacial flood waters cannot be directly observed until they break through the glacier terminus, this leads to an outstanding life-threatening challenge in flood early-warning.

Subglacial lakes and jökulhlaups (glacier outburst floods) occur in a range of settings and are common in volcanic environments where lakes are formed by geothermal activity or volcanic eruptions. It remains a challenge to determine the initiation of a flood event, quantify its size, and issue an early-warning as these environments are often remote and monitoring is hindered by ice cover. Warning times for civil defense response can therefore be short in the case of jökulhlaups, even in well hydrologically monitored areas. In the case of a jökulhlaup in Iceland in 2011 only 50 min elapsed between a warning from the local hydrological monitoring system and the flood reaching the country’s main transport artery, where it swept away a bridge^7^.

While the hydrological network can detect a flood reliably, it only detects it after the flood has progressed from beneath the glacier into the rivers. Methods that provide an earlier warning are therefore of important societal value. Whilst GPS instruments on the glacier surface above a subglacial lake could provide reliable and timely early-warning, a widespread use of this technology is challenging as maintaining GPS instruments in a harsh and heavily crevassed area above a subglacial lake is dangerous and costly and maintaining their telemetry from the ice surface is limited by snow accumulation during winter and electrical power considerations.

Seismic instruments have been found to be sufficiently sensitive and resilient to record ground vibrations associated with various moving sources; even when the instruments are tens of kilometers from the source. These sources range from landslides^8^, lahars^9^, to helicopters^10,11^. Here, constrained by exceptional multidisciplinary data, we determine how seismic ground vibrations in the form of tremor captured on specifically arranged arrays can be used to estimate key parameters associated with subglacial floods, including their size and propagation speed as they travel beneath the glacier. These new observations and interpretations could lead to more than an order of magnitude improvement in subglacial flood early-warning times.

## Results

### Seismic ground vibrations accompany subglacial floods

Bartholomaus et al.^12^ compared seismic tremor, subglacial discharge, and flow velocity of glaciers in Alaska and Greenland. While they reported no correlation between tremor and glacier motion, they found the best correlation with subglacial discharge for tremor frequencies between 1.5 and 10 Hz. Tremor amplitude varied with seasonal changes of the subglacial discharge but also on an hourly timescale during a subglacial flood.

This and other studies suggest that seismic tremor in glaciated regions is generated by turbulent flow in subglacial conduits and by transported sediment^12–16^. In other cases, tremor was related to englacial water flow in a moulin^17,18^, resonance in a water-filled crack or channel^17,19–21^ or repeating icequakes in moving or stationary icebergs^22–24^. For a detailed overview of glacial seismic sources such as icequakes and tremor the reader is referred to Podolskiy et al. (2016)^25^.

**Fig. 1:**
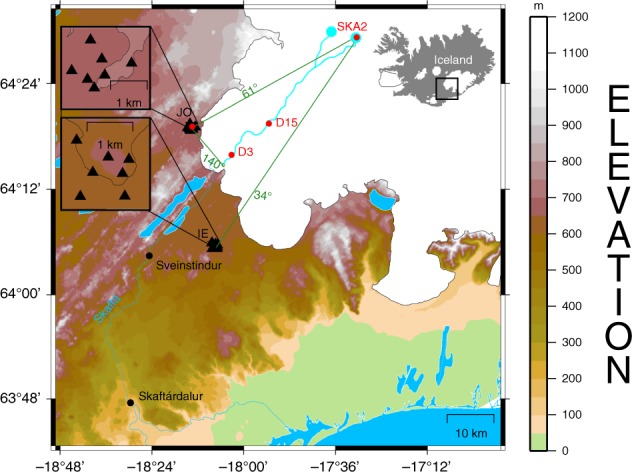
Overview of the multidisciplinary monitoring network around W-Vatnajökull ice cap. The eastern and western Skaftá cauldron (cyan dots), seismic arrays (black triangles), GPS instruments D3, D15 and SKA2 (red dots), hydrological instruments (black dots), subglacial flood paths as in Magnússon et al. (2004)^56^ (cyan lines on the glacier) and glacial rivers (cyan lines outside glacier) are marked. Back azimuths from JO and IE array to the eastern cauldron are indicated with green lines. The GPS instruments were installed for research purposes and are not part of the fixed monitoring network. The insets show an overview of Iceland and the geometry of JO and IE arrays in detail.

**Fig. 2:**
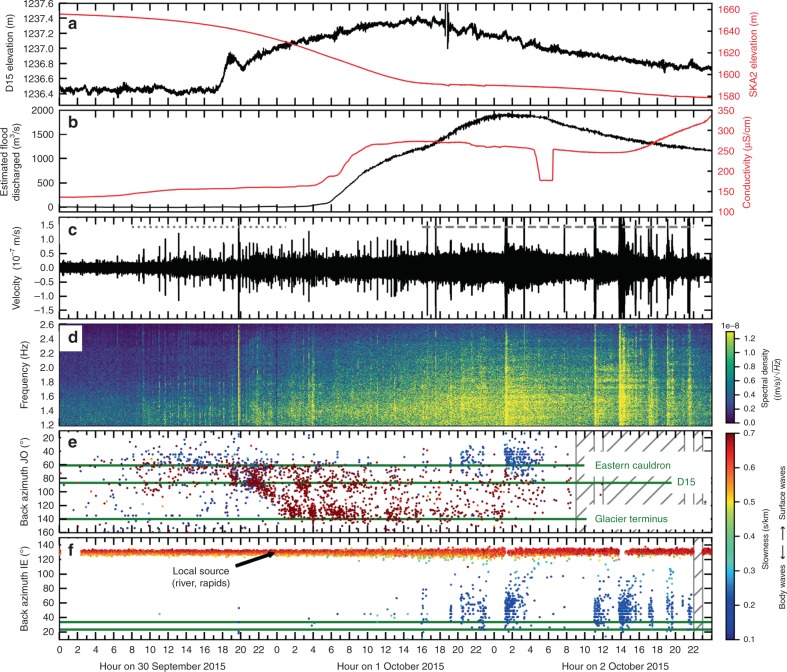
Tracking a large flood from the eastern Skaftá cauldron from 30 September to 3 October 2015. **a** Elevation of the GPS instruments on top of the cauldron (SKA2, red) and 15 km from the ice terminus (D15, black) as measured above the GRS80/WGS84 ellipsoid (67.23 m at D15 and 67.72 m at SKA2). **b** Conductivity (red) and estimated flood discharge (black) in Skaftá river measured at Sveinstindur. **c** Vertical velocity seismogram of JO station JOK, where the grey dotted and the grey dashed line mark time windows of Type 1 tremor and Type 2 tremor, respectively and (d) amplitude spectrogram of subfigure c made with a fast Fourier transform window length of 512 s and 50% overlap. **e** Dots indicate the dominant back azimuth at JO array coloured according to slowness. Green lines mark the direction of the eastern cauldron, the location of D15 and the glacier terminus outflow as in Fig. 1. Hatched times mark multiple hour long data gaps on more than four stations preventing array processing. The mean uncertainty caused by the array geometry is 4.2° (standard deviation 2.5°) in back azimuth and 0.04 s/km (standard deviation 0.01 s/km) in slowness. **f** Same as subfigure e but for IE array where green lines mark the back azimuth of the eastern cauldron and D15 (top and bottom, respectively). The mean uncertainty is 3.0° (standard deviation 1.1°) in back azimuth and 0.03 s/km (standard deviation 0.01 s/km) in slowness. Note that the recordings at IE array are dominated by local, surface wave noise known to be from nearby rivers and rapids (indicated by large slowness values). In comparison to this noise, Type 1 tremor is too weak to be detected by the array. However, the amplitude of the Type 2 tremor is larger than the local noise source and was recorded and located (indicated by small slowness values).

**Fig. 3:**
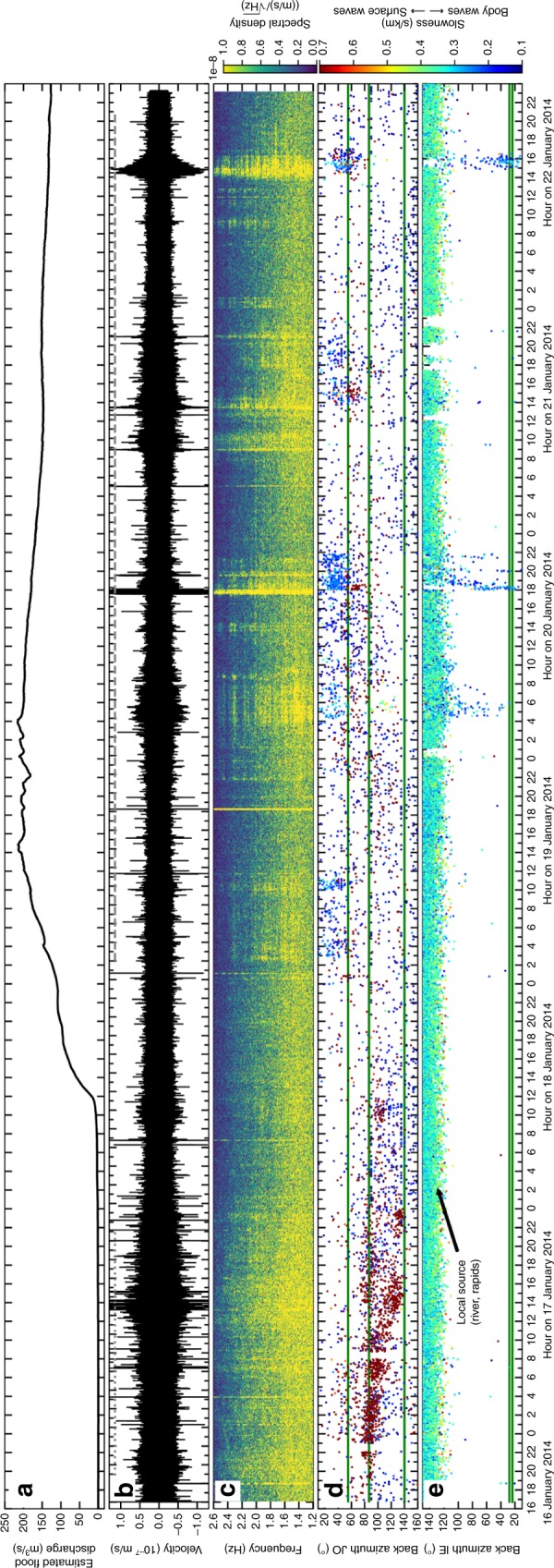
Tracking a small flood from the western Skaftá cauldron from 16 to 23 January 2014. **a**–**e** similar to Fig. 2b–f but for a different flood, which was not recorded by GPS data. **c** The spectrogram was created with a fast Fourier transform window length of 1024 s. **d** The mean uncertainty of each measurement is 4.9° (standard deviation 3.2°) in back azimuth and 0.04 s/km (standard deviation 0.01 s/km) in slowness. Green lines mark the direction of the western cauldron, the location of D15 and the glacier terminus outflow (top to bottom, respectively). **e** The mean uncertainty is 7.5° (standard deviation 1.7°) in back azimuth and 0.05 s/km (standard deviation 0.01 s/km) in slowness. The green lines mark the direction towards the western cauldron and D15 (top and bottom, respectively).

**Fig. 4:**
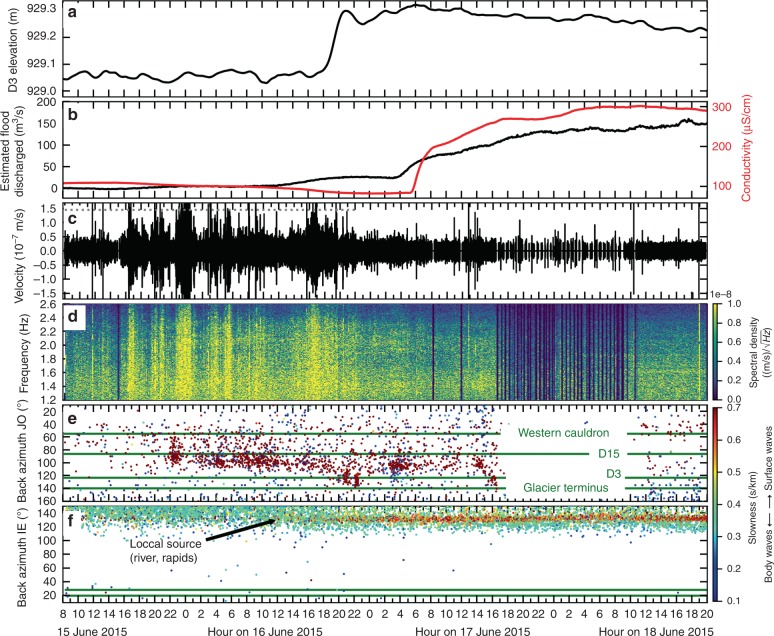
Tracking a small flood from the western Skaftá cauldron on 15–19 June 2015. All subfigures as in Fig. 2 with the following differences: **a** Elevation of GPS instrument at D3. **b**, **d** similar to Fig. 2b. **c** Note that seismic recordings at JO array contain small gaps starting 17 June due to data transmission problems. Time windows with a lot of gaps were mostly discarded from the array processing. For better readability only data gaps of several hours are hatched. **e** The mean uncertainty is 5.3° (standard deviation 2.8°) in back azimuth and 0.05 s/km (standard deviation 0.02 s/km) in slowness. **f** The mean uncertainty is 4.1° (standard deviation 2.4°) in back anazimuthd 0.04 s/km (standard deviation 0.03 s/km) in slowness. Green lines mark the direction towards the western cauldron and D3 (top and bottom, respectively).

Tremor was in the past also qualitatively linked to subaerial^15^ and subglacial floods in various glaciological, geomorphological or hydrological studies worldwide^20,26–30^. However, quantifying these relationships, to determine flood location, size or propagation speed has not yet been achieved. Here we quantify the links between ground vibrations and processes associated with a large (~3000 m^3^/s discharge) flood using seismic, geodetic and hydrological data and three smaller floods (210–380 m^3^/s peak discharge) (Figs. 1, 2, 3 and 4). We demonstrate that these ground vibrations yield first information on the initiation of the event and complement the early-warning currently gained from measurements of river height and electrical conductivity of river water^31^.

The seismic vibrations are also referred to as tremor or high-frequency noise based on the signals emergent start and long duration (Fig. 2c, d). Due to the lack of clear discrete onsets they cannot be located using traditional earthquake location methods. Instead, we use two clusters of seismometers (called arrays) consisting of seven seismometers each (six 3–component Güralp 6TDs (10 s to 100 Hz) and one 3–component Güralp 3ESPCD (60 s to 50 Hz)) (Fig. 1). In contrast to a seismic network, the arrays allow us to both locate the tremor source and determine the wave type in the tremor (surface vs. body waves) (e.g., Rost and Thomas (2002)^32^).

### Formation and drainage of subglacial lakes

The arrays were installed to study seismic tremor associated with floods originating in the eastern and western Skaftá cauldron (cyan dots in Fig. 1) in the southwestern part of Vatnajökull glacier. These two cauldrons are located in close proximity which is the reason why their subglacial flood paths are identical except for the uppermost 10 km. They generate regular floods, which enter the Skaftá river at the glacier outlet^3,33,34^.

The 1–3 km wide and 50–150 m deep^34^ Skaftá cauldrons are formed by geothermal activity. Small-scale, local melting of ice at the base of the glacier is followed by lake formation. The glacier surface depresses and circular crevasse patterns form around it. Consequently geothermal meltwater, geothermal fluids and percolating meltwater or precipitation (rainwater) within the watershed of the cauldron accumulate forming a subglacial lake^3,34,35^. The subglacial lakes drain along a  ~40 km long subglacial flood path when the pressure at the bottom of the lake is close to the ice-overburden pressure and a seal near the base of the ice fails^3,33,35^.

Floods occur every 1–5 years^33,36,37^ while typical discharges range from 50 to 3000 m^3^/s with total volumes of 0.05–0.4 km^3^. The floods are characterized by rapidly rising discharge curves^3,37^ where a propagating subglacial pressure wave^27,38^ is thought to create the initial pathway for sheet flow of water beneath the glacier^3,39^. Once a flood starts, they typically reach maximum discharge in 1–3 days and recede in 1–2 weeks^3^.

Note that this is in contrast to floods mentioned above that drain more slowly by melting channels into the ice such as reported by refs. ^12,16,30^.

### Tremor associated with different subglacial floods

From August 2013 to December 2016, three floods were released from the western Skaftá cauldron and one from the eastern Skaftá cauldron (for details see Table 1). Since the ice surface on top of the drained subglacial lake subsides, the flood origin can be determined from aerial photos of the area. The seismic recordings were fed into array processing^40,41^ as detailed in the “Methods”. The resulting angle between north and the direction towards the seismic tremor epicenter (back azimuth) and inverse of the apparent velocity (slowness) are plotted alongside seismograms and amplitude spectrograms in Figs. 2, 3 and 4. We can thus determine the direction to the source, the tremor source location and, from the slowness, determine the type of seismic waves comprising the tremor.

**Table 1:** Overview of total duration, the draining lake and the occurrence of Type 1 and Type 2 tremor during four floods from the eastern and western Skaftá cauldron in Iceland.

Total Duration	Draining	Type 1 Tremor	Type 2 Tremor	
of Flood	Lake	(non-harmonic, low frequency)	(harmonic, higher frequency)	
		(surface wave rich)	(body wave rich)	
16/01 – 30/01/2014	W Skaftá	16/01 18:00 to 17/01 21:00 + 3h	19/01 02:00 to 22/01 21:00	
15/06 – 30/06/2015	W Skaftá	15/06 08:00 to 16/06 22:00 + 19h	–	
30/09 – 04/10/2015	E Skaftá	30/09 08:00 to 01/10 01:20 + 7h	01 Oct. 16:00 to 02 Oct. 22:00	
06/09 – 20/09/2016	W Skaftá	06/09 0:00? to 07/09 08:00	–	

Type 1 tremor moved from the cauldron to the ice terminus outflow and persisted for some hours at the ice terminus (number following the ‘+’ sign).

Type 2 tremor did not propagate.

#### Eastern Skaftá cauldron

Two tremor types were observed during the flood from the Eastern Skaftá cauldron (Fig. 2) with peak discharge of 3000 m^3^/s in September to October 2015. We categorize them as Type 1 and Type 2 tremor as they are likely linked to different physical processes. Type 1 tremor initiates with the start of the sub-glacial flood. It is non-harmonic with broad frequency content, and a peak at 1.3 Hz. Its back azimuth changes as the sub-glacial flood progresses, demonstrating that the tremor is migrating. It is composed almost entirely of surface waves, indicating that it is generated near the Earth’s surface.

Changes in the continuity, harmonic character, amplitude and frequency content of the seismic signal, alongside changes in back azimuth and wave type indicate that type 2 tremor started. Type 2 tremor starts with a delay of more than one day after the flood initiates. It is stronger than Type 1 tremor and is composed of repeating, strong tremor episodes with frequency content up to 7 Hz and harmonic character, up to 8 h long. Its back azimuth does not change with time and points in the broad vicinity of the cauldron. It is composed almost entirely of body waves, indicating that it is generated at depth in the subsurface. Although we do discuss possible causes of Type 2 tremor, our primary focus here is on early-warning and hence on Type 1 tremor.

Since Type 1 tremor was emergent and the amplitude increased slowly, we used the array results to determine the start time of the flood. With increasing Type 1 tremor amplitude the semblance of the array output increased above our minimum semblance threshold and then starts to point in one direction rather than yielding scattered back azimuths. The onset of Type 2 tremor is distinct and can be determined directly from seismograms and spectrograms, and was confirmed by the array results. These floods are also accompanied by quakes, visible, for example, as peaks on 30 September in Fig. 2c.

Type 1 tremor persisted from about 08:00 on 30 September to 8:20 on 1 October 2015 (Table 1 and Fig. 2c–e) and showed a clear southwards movement with back azimuths increasing from 60 to 140°. In the same time window the GPS instrument on the ice surface above the subglacial lake indicated that water has already started to drain (red curve in Fig. 2a). The elevation of SKA2 started to decrease from noon on 27 September and accelerated in the early hours of 30 September, which we interpret as the time when the flood started to propagate. At 17:30 on 30 September the arrival of the propagating subglacial flood front is observed as lifting at a GPS station (D15), 15 km from the ice terminus and 28 km from the cauldron (black curve in Fig. 2a). At 04:00 on 1 October the flood water arrived at the hydrological instrument 25 km downstream in the Skaftá river (Fig. 2b). The GPS instrument D3 could not be used to detect the flood, as it was washed away when a small amount of the flood water reached the ice surface, hydrofracturing through the ice 3 km from the terminus.

**Fig. 5:**
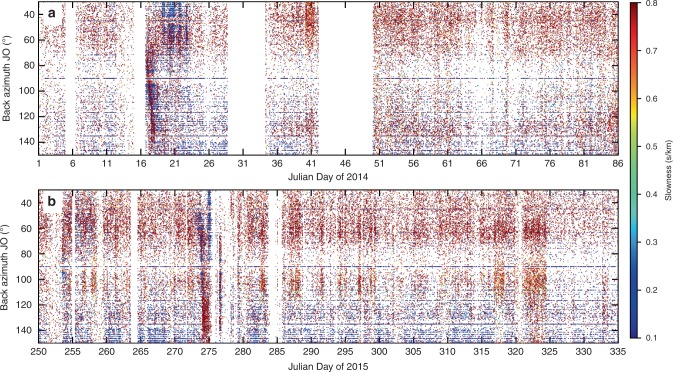
Back azimuth and slowness changes reveal subglacial floods. The seismic data were filtered 1.4–3.2 Hz. **a** Flood from Western Skaftá cauldron in January 2014; **b** Flood from Eastern Skaftá cauldron in September 2015. In the total window of 170 days, the systematically migrating back azimuth signal can only be seen at times of known floods (from day 17 and 273, respectively) and not at any other times. This allows the development of a robust causal relationship between systematic changes in tremor back azimuth away from the cauldron, and propagating floods. The two known floods were later determined by hydrological observation and visual evidence of flooding. Note that JO array has data gaps from Julian day 275 and therefore only shows the beginning of Type 2 tremor.

The propagation of the flood derived from GPS and hydrological instruments is consistent with the systematically increasing back azimuth determined for the tremor based on seismic array analysis. To alleviate any doubt about the origin of the migrating tremor signals in Fig. 2e and to demonstrated that they are unequivocally related to floods, in Fig. 5 we show back azimuth plots for two 85-day long time windows that each include known floods, as subsequently identified by water discharge. The back azimuth and slowness signals have uniquely distinctive features only at times of the known flood, and not at any other times. We have visually inspected 3.5 years of data and find that the systematically increasing back azimuths seen in Fig. 5 are unique to known times of sub-glacial floods as subsequently determined by other observables. We therefore conclude that the moving tremor source tracked the subglacial propagation of the flood front and hence can be used as an early-warning tool, detecting the flood ~17.5 h before its front reached the ice terminus. The flood propagated from the ice terminus to the uppermost hydrological station in ~2.5 h, arriving at this hydrological station 20 h after it was first detected in the seismic wavefield as Type 1 tremor.

Type 2 tremor started from the direction of the cauldron area at 16:00 on 1 October. This is about 14.5 h after the flood front reached the ice terminus and Type 2 tremor continued for 30 h (Fig. 2c–f). The GPS instrument on the ice surface above the lake indicated (red curve in Fig. 2a) that at that time most water had left the lake. However, for about 30 h the lowering of the ice surface decelerated which is interpreted as the time period when some parts of the roof of the subglacial cavity reached the bedrock. When the subsidence of the roof of the subglacial cavity at the location of the GPS instrument SKA2 stopped, the Type 2 tremor stopped as well. Note that since Type 2 tremor was an order of magnitude stronger than Type 1 tremor, a high tremor amplitude was recorded in the early hours of 2 October, when the flood peak had already reached the hydrological station 25 km downstream. Hence, despite its strength Type 2 tremor does not give an early-warning advantage and does not migrate spatially. Advanced early-warning can only be seen in the weaker migrating Type 1 tremor.

Type 2 tremor is characterised by slownesses of 0.1–0.2 s/km (Fig. 2e, f). Eibl et al.^42^ located earthquakes originating in the southwestern Vatnajökull region and analysed the slownesses recorded at JO and IE arrays associated with P-, S- and surface waves. They concluded that typical surface wave slownesses for this array configuration are above 0.7 s/km, while typical body wave slownesses are around 0.15 s/km. We therefore conclude that Type 2 tremor is almost exclusively composed of body waves, but cannot distinguish between P- and S-waves as their respective apparent slownesses across the array are too similar. In contrast to Type 1 tremor, Type 2 tremor is composed of body waves and therefore is likely generated in the bedrock in the region of the draining subglacial lake.

In summary Type 1 tremor is associated with and can be used to track the propagating subglacial flood front. Type 2 tremor coincides in time with a large pressure drop beneath the cauldron. Its cessation may indicate that the roof of the subglacial cavity has subsided to its equilibrium position, following a draining episode (see “Discussion”).

#### Western Skaftá cauldron

A flood with peak discharge of 380 m^3^/s^43^ from the Western Skaftá cauldron was recorded in January 2014. Due to a data gap on 15 January we can merely state that Type 1 tremor started at least 34 h before the flood reached the uppermost hydrological instrument (Fig. 3). A clear southwards propagation of the tremor source is visible for about 23 h, interpreted as the subglacial propagation of the flood. About 29 h after the flood front reached the ice terminus harmonic, 2–9 h long Type 2 tremor from the cauldron area with energy up to 10 Hz started and persisted for 3.5 days. The tremor during the western Skaftá flood in January 2014 resembled the tremor during the eastern Skaftá flood in September 2015, but Type 1 tremor propagated more slowly and Type 2 tremor persisted three times longer and was of lower amplitude.

Two further smaller floods from the western Skaftá cauldron in June 2015 (210 m^3^/s peak discharge) (Fig. 4) and September 2016 (330 m^3^/s peak discharge) were accompanied by weaker Type 1 tremor and no subsequent Type 2 tremor. The propagation of the flood front was visible in about 38 hours of moving Type 1 tremor on 15–16 June 2015 (Fig. 4), and in 34 hours of moving Type 1 tremor on 6 and 7 September 2016.

The propagation of a subglacial pressure wave is observed as a lifting of 0.25 m at the on-glacier GPS station D3, 3 km from the ice terminus around 17:00 on 16 June 2015 (black curve in Fig. 4a). The flood emerged from beneath the glacier and reached the hydrological station at 04:00 on 17 June. The propagation of the flood front derived from GPS and hydrological instruments is, therefore, consistent with the systematically increasing back azimuth determined from the tremor. This is in agreement with our observations for the large flood from the eastern cauldron in October 2015.

## Discussion

### Flood magnitude and timing in flood early-warning

**Fig. 6:**
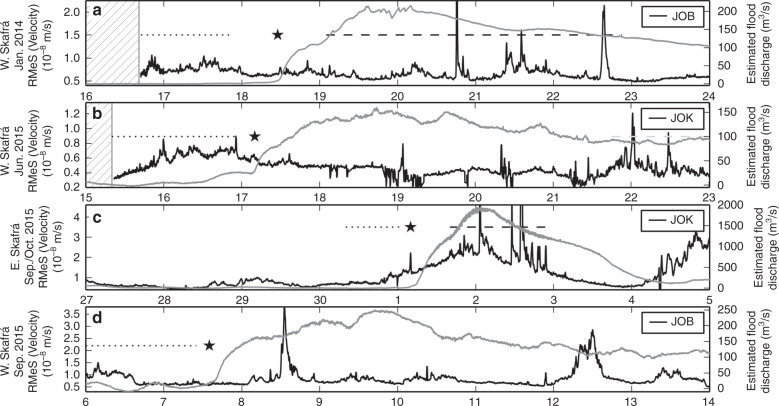
Summary of tremor and estimated discharge during all four floods. The floods occurred in **a** January 2014, **b** June 2015, **c** September/October 2015 and **d** September 2016. Hatched areas mark data gaps. Root Median Square (RMeS) (black) of the seismic ground motion recordings at station JOK or JOB in JO array was filtered between 1.3 and 4 Hz. The station with the least data gaps is shown alongside the estimated flood discharge (grey). The star marks the arrival of the flood at the uppermost hydrological station. Dotted lines mark the time period when the tremor source is propagating southwards using JO array during Skaftá floods (Type 1 tremor), dashed lines mark time periods of high-frequency tremor with harmonic character (Type 2 tremor).

An overview of tremor amplitude and discharge (Fig. 6) comprises 8 day long time windows around the four floods from the eastern and western Skaftá cauldron. For clarity and in order to enhance the tremor and discard shorter transients, such as earthquakes^42^, we display Root Median Square (black) tremor amplitudes at one station in JO array. This is complemented by the discharge measured at the hydrological station 25 km downstream (grey). Based on Figs. 2e, 3d and 4e we have also marked the times when Type 1 tremor or Type 2 tremor persisted, with dotted and dashed lines, respectively. Note that the tremor in January 2014 and September 2016 is weak and barely visible in the RMeS. This and its non-harmonic character demonstrates the need for an array to locate and track the moving tremor source. A simple spectral analysis of the seismic data recorded on the standard seismic network is not sufficient.

**Fig. 7:**
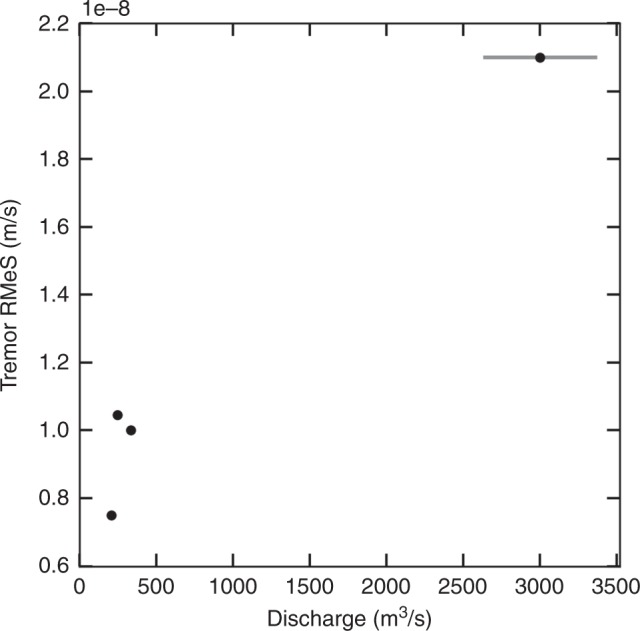
Peak discharge and Type 1 tremor amplitude correlate. Peak discharge during floods from the Skaftá cauldrons in comparison to peak Root Median Square (RMeS) tremor amplitude during the subglacial propagation of the flood front from the lake to the ice terminus (Type 1 tremor). Contamination of the peak amplitude by short transients such as earthquakes is avoided by calculating RMeS instead of Root Mean Square of the seismic waveform and by focussing on the time window where Type 1 tremor is present. Error bars on discharge values below 1000 m^3^/s and tremor are smaller than the symbol used. Uncertainty for higher discharge values was estimated from the combined uncertainty of the discharge measurements at Sveinstindur and the uncertainty on the estimate of discharge that bypasses the hydrometric station^52^.

Despite flood sizes ranging over an order of magnitude (Fig. 7), all four floods from the Skaftá cauldrons created southwards moving Type 1 tremor signals detectable at more than 10 km distance. This tremor is composed of surface waves which is consistent with^16^ who recorded tremor comprising surface waves at more than 1 km distance from the source caused by subglacial water flow. It is apparent from Fig. 6a–d that the smallest Skaftá flood (June 2016) was accompanied by the weakest Type 1 tremor, while the largest flood generated the strongest tremor. We suggest that the tremor amplitude can therefore be used as a proxy for how large the flood will be, while it is still travelling beneath the ice.

The progressive lifting and increased horizontal motion of the glacier initiated by the pressure wave leads to fractures at the base of the ice. A multitude of these icequakes or quakes at the interface of bedrock and ice may merge into the continuous signal that we detect as tremor. However, fluid motion at the base of the glacier or transported sediment are alternative possible mechanisms for tremor generation. Here we focus on the positive correlation between tremor amplitude and discharge (Fig. 7), which is in accordance with the published literature^12,13^. In contrast to these studies, we cannot however produce such a tight similarity between the shape of the discharge and tremor curves in time. We note that although a clear positive correlation between tremor amplitude and discharge is evident, the relationship here for sheet flow is not as well defined as in R-channel flow (see e.g., Bartholomaus et al. 2015^12^).

Given that there is no GPS instrument in the western cauldron and the hydrological station 25 km downstream cannot be used to precisely time the emergence of the flood at the ice terminus, we rely on the seismic recordings in order to calculate the average speed of all four floods. Based on array recordings of Type 1 tremor, it is possible to estimate the speed of the flood front. The subglacial flood from the eastern Skaftá cauldron in 2015 moved 25–42 km (back azimuths of 60–68° to 140°) in 17.5 h and therefore at an average speed of 1.4–2.4 km/h (0.4–0.7 m/s). According to the GPS recordings the floods travelled 12 km from D15 to the glacier terminus outflow at a speed of ~2.0 km/h on the lowermost part of the subglacial flood path. The tremor sources during the flood in January 2014 propagated at 0.9–1.6 km/h, the one in June 2015 at 0.7–1.1 km/h and the one in September 2016 at 0.8–1.3 km/h. These are upper limits of the velocity due to data gaps in the seismic recordings at the beginning of the floods in January 2014 and June 2015. An order of magnitude reduction in flood size led to a 50% reduction in flood propagation speed and in tremor amplitude (Fig. 7).

The challenge to distinguish between volcanic and flood-related tremor in glaciated volcanic areas is long standing. Studying tremor associated with floods of various sizes gives us the opportunity to determine typical flood-related characteristics. These are for example that Type 1 tremor is diffuse and strongest around 1.3 Hz. Floods can best be tracked seismically using an array, and can be distinguished from a magmatic source through their propagation speed. During the Bardarbunga-Holuhraun eruption the dike propagated at an average speed of 4.4 km/day at depth^42^ while lava flowed on the surface at a speed of 0.1–1.3 km/day^44^. Therefore, magma flowed more than 13 times more slowly than the subglacial floods.

The practical application of the observations goes beyond merely distinguishing between magma or lava movement and sub-glacial floods. The ability to track the direction and speed of flood propagation can serve as a subglacial flood early-warning tool. However, unequivocal determination of the initiation time of the propagating flood does require some careful considerations (see section “Practical applications and outlook” in “Discussion”).

Array analysis results demonstrate that the Type 2 tremor was composed of body waves and was located at or close to the draining cauldron. Tremor episodes with similar spectral content and shape of the mean amplitude per minute recorded at a single seismic station were reported at the end of floods from a subglacial lake at Grimsvötn^26^ and a subaerial lake at Kverkfjöll^45^. Bodvarsson et al.^26^ stated that the Type 2 tremor was up to 30 min long and visible on most seismic stations in Iceland. They suggest that a sudden pressure drop due to the emptying of the cauldron led to boiling in the subglacial geothermal field. Montanaro et al.^45^ studied hydrothermal explosions at Kverkfjöll combining field work, laboratory studies, seismic data and analytical models. A flood from a lake led to a lake level drop of 30 m and hydrothermal explosions. The seismic characteristics were surface wave-dominated, 40–50 s long energetic tremor episodes with energy up to 4 Hz and most energy between 0.5 and 2.5 Hz at a station at 19 km distance. They interpreted these several minute long, harmonic tremor episodes as hydrothermal explosions and vigorous boiling and found that it located in an ice-free area. Other generation models for our Type 2 tremor such as a swarm of basal icequakes or basal motion at the bottom of the glacier (e.g., Lipovski et al. 2016^46^) seem unlikely due to their similarity to tremor signals generated in an ice-free area. In addition, in this study Type 2 tremor is dominated by body waves and therefore is unlikely to be generated solely at the Earth’s surface.

We observed that Type 2 tremor started in a time period where most water had drained from the cauldron. It ceased when the roof of the subglacial cavity had settled back on the bedrock. In this time window the geothermal system underwent a pressure drop and due to spectral similarities to the tremor at Kverkfjöll (duration, broad frequency content and harmonic character) we follow the interpretation of Montanaro et al.^45^ that a likely source mechanism for the tremor are hydrothermal explosions and boiling of the geothermal system in the rocks underlying the subglacial lake, triggered by a pressure drop.

We suggest that a second phase of conductivity increase, starting on 2 October 2015 (Fig. 2b) indicates an increased amount of suspended material in the flood water, injected into the subglacial lake from the geothermal system by the explosions/boiling events. Such second phase conductivity increases are common in jökulhlaups from the Skaftá cauldrons and have been interpreted in this manner before^28,47^. The conductivity increase is higher than can be expected from other non-geothermal sources. Anderson et al.^48^ used an increase in solute concentration during the waning phase of a jökulhlaup from Hidden Creek lake in Alaska to infer a release of long residence time water from the distributed subglacial hydraulic system due to lowering of water pressure in the flood path. This is an unlikely explanation for the conductivity increase in the Skaftá floods, as such water, released from the glacier in late winter, has been observed to have conductivity below 200 μS/cm (unpublished data of the Icelandic Meteorological Office) and would have caused a lowering in the observed conductivity.

The lack of detectable Type 2 tremor in June 2015 and September 2016 is unresolved but could be related to slower or insufficient pressure decrease in the subglacial lake, for these smaller floods. The absence of explosions/ boiling in June 2015 is consistent with the absence of a second phase of increase in conductivity (Fig. 4). Reliable conductivity data are not available for the January 2014 and September 2016 floods due to sediments clogging the sensor.

### Practical applications and outlook

Jökulhlaups can be categorized as part of a spectrum of floods from rapidly rising where the initial flood path is mainly formed by lifting and deformation of the glacier due to a propagating subglacial pressure wave, to slowly rising where the rise in flood discharge is controlled by the melting of conduits or channels at bottlenecks in the flood path^3^.

Here, we studied four rapidly rising floods that were in the past mainly monitored and detected using hydrological instruments 25 km downstream in the affected river once the flood emerged from beneath the glacier. An earlier warning is desirable and of high societal value and can be given using GPS instruments on the ice immediately above the expected flood path or above the subglacial source lakes. However, multiple subglacial lakes, dangerous installation and maintenance work in a heavily crevassed area, snow accumulation, technical problems with the telemetry of the data, limited power generation, or the vulnerability of sensors to being washed away if part of the flood water reaches the ice surface all limit this application for practical purposes.

We show that seismic arrays allow the continuous tracking of a subglacial flood front and its propagation speed. Here we demonstrate that subglacial floods in southwest Vatnajökull, Iceland, are accompanied by two different types of seismic ground vibrations that contain key information about the flood and can complement hydrological measurements leading to advanced flood warnings.

Type 1 tremor is non-harmonic, low frequency and rich in seismic surface waves, while Type 2 tremor is harmonic, of higher frequency and rich in body waves. Type 1 tremor was in all cases detected more than 20 h before the hydrological station recorded an increase in water height and could be used to track the movement of the front of floods with peak discharges ranging from around 3000 m^3^/s down to at least 200 m^3^/s. Moving back azimuths obtained through array analysis of the signal indicate the speed of the flood. At later stages half of the floods that we observed are followed by Type 2 tremor generated in the area of the draining subglacial lake. This tremor can be explained by explosions/ hydrothermal boiling events that are triggered by the pressure decrease on the geothermal system.

The southernmost seismic array (IE) detects an area of rapidly flowing water in a local river to the south east of the array (see Fig. 2f). Tremor levels are seen to change with flow rate throughout the seasons^49^. This indicates that array detected tremor at this site can be directly related to water flow. Although sub-glacial processes are more complex than river flow, comparing on-glacier GPS vertical displacement and seismic tremor timings suggests that the sub-glacial tremor is primarily generated at the flood front. This ability to detect water flow implies that the strength of the sub-glacial tremor and its propagation speed may directly indicate the size of the impending flood. Applying real-time data analysis would allow early-warning estimates of both flood size and flood arrival times at the ice-terminus.

The practical application of these observations as a flood early-warning tool depends on the ability to reliably detect systematic changes in the back azimuths that lie above the scatter associated with individual measurements. The sensitivity of such a system depends on the location of the array with respect to the draining water source. Ideally it should detect weak signals at the beginning of a flood and be able to resolve tremor source location changes (migration) as a large change in back azimuth. For the floods analysed here, the slow initial change in back azimuth is due to the network geometry and is not a source characteristic. We need at least a 12 h long time window in order to unambiguously resolve flood movement. Critical points are the distance between the array, the flood source region that affects the amplitude of the signal, and the azimuth of the array relative to the flood movement direction. In an ideal array configuration the array would be both close to the initiation point of the flood and perpendicular to the flood propagation direction.

In the case presented here for the JO array, it is 40 km from the flood source region at an angle of ~15° to the initial flood path direction. Consequently, although Type 1 tremor signals can clearly be seen at flood initiation (up to 20 h before the flood is detected in the Skafta river), it does not appear to migrate at first due to this geometric configuration. However, a conservative estimate based on an examination of Fig. 2e demonstrates that unequivocal back azimuth changes associated with the propagating flood front can be seen at least 8 h before the flood is detected at the hydrological station. Hence, in this example, in practice an Observatory team would have heightened awareness of increased tremor coming from the cauldron 20 h in advance, and may wish to issue an early-warning. Alternatively a conservative approach focused on avoiding false alarms would lead to unequivocal indications that a flood was in play at least 8 h ahead of current hydrological-based early-warning capability. Hence even when being conservative this is a substantial amount of additional warning in terms of risk mitigation in the region. We suggest that the same advantage would apply to other regions in the world, taking the same approach.

Subglacial floods were also reported in non-volcanic environments such as Greenland and Antarctica^12,20^. Bartholomaus et al.^12^ showed that the tremor amplitude correlated with long-term variations in the subglacial discharge as well as short-term variations due to a slowly-rising flood with a delay in the tremor amplitude of a few hours. Our findings for fast-rising and ice-sheet lifting subglacial floods in volcanic environments show that flood size, location and speed can be determined as well and can be adapted to other environments worldwide. We envisage that in the future arrays located at a location from which the observed back azimuths during the flood are almost perpendicular to the flood path, will allow the resolution of the flood speed continuously along the whole path. We demonstrate that array-detected seismic tremor represents a new quantitative tool for early-warning during subglacial floods in various flooding environments and has the potential to give advanced warning of flood arrival-time, size and expected duration.

## Methods

### Seismic network

Two seismic arrays were installed southwest of Vatnajökull glacier around Skaftá river in order to monitor floods from the eastern and western Skaftá cauldron (see Fig. 1). The arrays consist of seven 3-component seismometers each (6 Güralp 6TDs (10 s to 100 Hz) and 1 Güralp 3ESPCD (60 s to 50 Hz)), have an aperture of 1.63 km and a minimum station spacing of 360 m. Their geometry was designed for tremor frequencies in the range of 0.4–6 Hz assuming typical P wave velocities of 2.5 km/s^50^ in the volcanic zone of Iceland.

We instrument correct, detrend, downsample the data to 20 Hz and cut it into 1 h long time windows before performing an array processing between 1.4 and 3.0 Hz as implemented in refs. ^40,41^. A shorter moving time window of 35 periods in length (66.5–87.5 s) and 20% overlap is used to perform a grid search in a horizontal slowness grid (stepsize 0.02 s/km and limit of  ±1.0 s/km). Based on the coherence of waveforms of the array stations, the array processing determines a time series of absolute power, semblance, back azimuth and slowness. The back azimuth gives the direction between north and the incoming seismic wave, the slowness is the inverse of the apparent seismic wave velocity through the ground and contains information about the steepness of the incoming wave and therefore the wave type (e.g. surface or body waves). We require a minimum semblance of 0.3 and determine the uncertainty in back azimuth and slowness based on the location and shape of the main lobe of the array response function. The back azimuth and slowness of all points with a power of at least 95% of the maximum are determined and their standard deviation is given as uncertainty. All points with uncertainties above 12° in back azimuth and 0.2 s/km in slowness are discarded.

Additionally and completely independent of the array processing, we calculate the Root median square (RMeS) of the seismic amplitude in order to assess the tremor amplitude and give less weight to shorter transients such as earthquakes^42^. We instrument-corrected, detrended, tapered and filtered the vertical component of the seismic recording between 1.3 and 4.0 Hz. We divided the seismic recording into 15 min long time windows and calculated the root median square of the signal. We repeated this iteratively allowing 20% overlap.

### GPS and hydrological network

The discharge of the jökulhlaups in Skaftá was measured by the Icelandic Meteorological Office (IMO) at an hydrometric station at Sveinstindur, 25 km downstream of the glacier margin (Fig. 1). The discharge was measured by continuously monitoring the water-level in the river, using a pressure transducer located upriver from a stable natural controlling cross section. The water level is converted to discharge using a water-level-discharge rating curve that is constructed from discrete water-level and discharge measurements. The uncertainty of discharge measurements at Sveinstindur is estimated at ±2% for discharges below 1000 m^3^/s^51,52^. Part of the river discharge bypasses the hydrometric station in large floods such as the September to October 2015 flood. This increases uncertainty in measured flood discharge substantially and explains difference in estimated maximum of ~3000 m^3^/s (Fig. 7) and measured flood discharge (Figs. 2 and 6) with maximum of ~2000 m^3^/s^52^. The total uncertainty of the maximum discharge estimate was estimated as ±360 m^3^/s from the combined independent uncertainty of the discharge measurements at Sveinstindur (±2%) and the uncertainty on the estimate of discharge that bypasses the hydrometric station (±350 m^3^/s)^52^. Base flow in the river, due to sources other than the jökulhlaup’s flood water, is estimated using the hydrological model WaSiM^53^ and subtracted from the discharge measured at the hydrometric station. This further increases the uncertainty of estimated flood discharge by ±12 m^3^/s^47^.

Electric conductivity of the river water is measured at the hydrometric station at Sveinstindur. Increased conductivity is used as an indication of flood water in the river^31^, as the dammed water in subglacial lakes over geothermal areas has higher amount of total dissolved solids than water from other runoff sources^54^. The sensor often gets clogged with sediments, which can make the measurements unreliable as is the case for the January 2014 and September 2016 floods.

In 2015 a streaming GPS instrument was maintained by the IMO in the eastern Skaftá cauldron (SKA2) and two GPS instruments were installed on the ice surface above the flood path on Skaftárjökull (Fig. 1). The stations on Skaftárjökull are denoted as D3 and D15 based on their distance from the glacier margin. The instrument in the cauldron was installed on a pole drilled into the ice while the instruments above the flood path were installed on top of the glacier surface on quadropods. Elevation changes observed by the instruments on the quadropods are affected by lowering due to surface melting, and by the local glacier slope and the down-glacier movement of the ice. These processes are not corrected for as rapid lifting during the arrival of a propagating flood front is on the order of tens of centimeters while these effects are on the order of centimeters per day. The GPS instruments used were Trimble NetRS dual-frequency receivers recording at 15 s intervals. The GPS data were processed with the GAMIT-Track utility^55^ with the continuous GPS station at JO (Jökulheimar, length of baselines ~9–40 km) as base. The standard deviation of unfiltered positions around a daily mean during periods of slow motion and low melt is lower than 4 cm in the vertical coordinates and may be used as an indication of the precision of the GPS measurements. GPS measurements are not available from D15 in the June 2015 jökulhlaup due to power problems, and neither from D3 in the September to October 2015 jökulhlaup as the instrument was washed away when a small part of the flood reached the ice surface by hydrofracturing the ice 3 km from the terminus.

## Data Availability

Seismic data are available via the website: http://futurevolc.vedur.is/.

## Code availability

Data was processed using the freely available software Obspy. For inquiries about the code please contact E.P.S.E. at eva.ps.eibl@hotmail.com.
