# Publisher Correction: Seismic ground vibrations give advanced early-warning of subglacial floods

**DOI:** 10.1038/s41467-020-17624-4

**Published:** 2020-07-21

**Authors:** Eva P. S. Eibl, Christopher J. Bean, Bergur Einarsson, Finnur Pàlsson, Kristin S. Vogfjörd

**Affiliations:** 1 Institute of Geosciences, University of Potsdam, Potsdam, Germany; 2 School of Earth Sciences, University College Dublin, Belfield, Dublin Ireland; 3 Geophysics Section, School of Cosmic Physics, Dublin Institute for Advanced Studies, Merrion Square, Dublin Ireland; 4 Icelandic Meteorological Office, Bústaðavegi 7–9, 108 Reykjavík Iceland; 5 Institute of Earth Sciences, University of Iceland, Askja, Building of Natural Sciences, Sturlugata 7, 101 Reykjavík Iceland

**Keywords:** Natural hazards, Geophysics, Seismology

Correction to: *Nature Communications* 10.1038/s41467-020-15744-5, published online 19 May 2020.

The original version of this Article contained an error in Fig. 6, in which the *y*-axis label of panel (c) was mislabelled as ‘W. Skaftá’. The correct *y*-axis label of panel (c) is ‘E. Skaftá’. This has now been corrected in the PDF and HTML versions of the Article.

